# Clinical features and outcomes of retroperitoneal unicentric Castleman disease resected as sarcomas: insights from a high-volume sarcoma center

**DOI:** 10.3389/fsurg.2024.1371968

**Published:** 2024-09-05

**Authors:** Haicheng Gao, Wenjie Li, Boyuan Zou, Shibo Liu, Chengli Miao

**Affiliations:** Department of Retroperitoneal Tumor Surgery, Peking University International Hospital, Beijing, China

**Keywords:** unicentric Castleman disease, retroperitoneal, surgery, complications, prognosis

## Abstract

**Background:**

Castleman disease (CD) is a rare lymphoproliferative disorder that can occur anywhere along the lymphatic pathway. Retroperitoneal unicentric Castleman disease (UCD) is an extremely rare manifestation. This study aims to explore the clinical features and surgical treatment of retroperitoneal UCD.

**Methods:**

We retrospectively reviewed patients who underwent retroperitoneal tumor surgery and were diagnosed with CD based on postoperative pathology before December 31, 2022. Data from these patients were collected and analyzed.

**Results:**

A total of 15 patients were included in the final analysis. All patients underwent radical resection under general anesthesia. Two out of 15 patients (13.3%) experienced serious complications but recovered well. There were no perioperative deaths. The median follow-up time was 78.5 months (range: 18–107.5 months), and no deaths or recurrences occurred during this period.

**Conclusions:**

Surgical treatment for retroperitoneal UCD is safe. Patients with retroperitoneal UCD can achieve long-time survival through complete resection.

## 1 Introduction

Castleman disease (CD) comprises a group of heterogeneous disorders involving lymphoid tissue and is considered very rare. Based on the number of lymph node stations involved, CD can be classified into unicentric CD (UCD) and multicentric CD (MCD) (1, 2). Histologically, CD can be further divided into hyaline-vascular, plasma cell, and mixed types. However, our understanding of the epidemiology and etiology of CD remains limited (3, 4).

The treatment and prognosis of MCD are complex and significantly differ. Although consensus exists that surgical resection should be considered for UCD patients whenever feasible, managing UCD occurring in the retroperitoneum remains challenging (5–7). The deep location, complex surrounding organs, and blood vessels make surgical treatment of retroperitoneal UCD high-risk. Additionally, distinguishing this disease from primary retroperitoneal sarcomas based on imaging examinations (such as lymphoma, leiomyoma, and paraganglioma) poses difficulties (8–12).

As a high-volume center specializing in retroperitoneal sarcoma treatment, we observed that some patients initially diagnosed with retroperitoneal tumors were pathologically confirmed to have CD after surgery. Given the rarity of this disease, we conducted a retrospective analysis of retroperitoneal CD patients treated in our center to gain insights into the disease's characteristics, treatment strategies, and prognosis.

## 2 Materials and methods

### 2.1 Patient selection

We retrospectively reviewed patients treated at our center from January 1, 2014 to December 31, 2022. Among them, 20 patients had a definitive pathological diagnosis of Castleman disease (CD), confirmed either by needle biopsy or surgical resection. Exclusion criteria included patients with a history of other malignancies, those who underwent needle biopsy only and declined surgery, and one patient diagnosed with multicentric Castleman disease (MCD) after thorough examination. Ultimately, 15 patients with retroperitoneal unicentric Castleman disease (UCD) were included in the final analysis.

### 2.2 Imaging and diagnostic criteria

All patients underwent contrast-enhanced computed tomography (CT) scans of the neck, chest, abdomen, and pelvis, or ultrasound examinations of the involved regions/organs and superficial lymph nodes. Additional systemic positron emission tomography (PET) scans were performed as needed. UCD was defined as a solitary site of mass without other suspicious lesions.

### 2.3 Data collection and statistical analysis

We established a comprehensive database from medical records, including patient demographics (gender, age), body mass index (BMI), presenting symptoms, blood test results, radiological lesion size, and pathology subtype. Surgical details, such as the surgical approach, operative time, estimated blood loss, length of postoperative stay, and postoperative complications, were also recorded. Patients were followed up via telephone conversations, with the last follow-up date set at November 1, 2023. The primary endpoint was disease-related death or disease recurrence. Survival analysis was conducted based on the occurrence of endpoint events during follow-up.

Categorical variables were expressed as frequencies and percentages, while continuous variables were presented as means with standard deviation (SD) or medians with ranges or interquartile ranges (IQRs), depending on the distribution normality. Data analyses were performed using SPSS v22 statistical software (Chicago, IL, USA).

### 2.4 Ethics approval and informed consent

This study adhered to the ethical standards outlined by the responsible committee on human experimentation (both institutional and national) and followed the principles of the Declaration of Helsinki (1964 and subsequent revisions). The Institutional Review Board of Peking University International Hospital approved the study, and informed consent (or an appropriate substitute) was obtained from all patients before their inclusion.

## 3 Results

### 3.1 Clinical features

The clinical characteristics of 15 retroperitoneal UCD patients are summarized in Table 1. The ratio of male to female patients was 2.75:1.00. The median age was 31 years (range, 24–58 years), with 80% patients younger than 40 years. The histology subtype was hyaline-vascular for 10 patients (66.7%), mixed type for 5 patients (33.3%), and 0 plasma cell type. B symptoms (fever, night sweats, and weight loss) were present in 2 patients (13.3%). Pleural effusion was found in 1 patient (6.7%). Splenomegaly was found in 1 patient (6.7%). Most lesion sizes were smaller than 10 cm (6.9 ± 2.3 cm) (showed in Table 1). The laboratory tests were generally normal for all patients, including blood routine examination, serum biochemical indicators, C-reactive protein and renal function (showed in Table 1). Only one patient had suspected TAFRO syndrome, with splenomegaly and pleural effusion.

**Table 1:** Clinicopathological characteristics of 15 patients with retroperitoneal unicentric Castleman disease.

Item	Number	Proportion (%)
Age (years)		
<40	12	80
≥40	3	20
Gender		
** **Female	4	26.7
** **Male	11	73.3
B symptoms	2	13.3
Ascites and/or pleural effusion	1	6.7
Splenomegaly	1	6.7
Pathology		
HV	10	66.7
Mix	5	33.3
PC	0	0
Item	IQR/mean ± SD	
HGB (g/L)	142 ± 26	
WBC (10^9^/L)	4.93 ± 0.65	
Platelet (10^9^/L)	209 (156, 268)	
Albumin (g/L)	42.97 ± 5.71	
CRP (mg/L)	1.27 (0.86, 2.40)	
eGFR (ml/min)	115.98 ± 16.10	
Size (cm)	6.9 ± 2.3	

HV, hyaline-vascular; PC, plasma cell; HGB, hemoglobin; WBC, white blood cell count; CRP, C-reactive protein; eGFR, estimated glomerular filtration rate; IQR, interquartile range.

### 3.2 Surgical details

Patients were admitted to hospital for retroperitoneal lesions suspected of malignant sarcomas. The surgical strategy aimed for radical resection with adjacent tissue dissection, ensuring negative margins.

Thirteen patients underwent traditional open surgery, while two patients received laparoscopic surgery. The average operation time was 186 min (186 ± 57 min). Estimated intraoperation blood loss ranged from 50 ml to 1,500 ml, and median volume was 400 ml (showed in Table 2). Two patients experienced severe postoperative complications and recovered well after treatment (details in Table 3). All patients were discharged with satisfactory recovery. There were no perioperative deaths or readmissions within 30 days. The average length of stay after surgery was 11.2 days (11.2 ± 3.6 days).

**Table 2:** Details of surgical treatment for 15 retroperitoneal unicentric Castleman disease patients.

Item	Number	Proportion (%)
Approach		
Open	13	86.7
LAP	2	13.3
Postoperative complications (Grade III/IV)		
Yes	2	13.3
No	13	86.7
Item	IQR/mean ± SD	
Operation time (min)	186 ± 57	
Estimated blood loss (ml)	625 ± 477	
LOS (day)	11.2 ± 3.6	

LAP, laparoscope; LOS, length of stay after operation.

**Table 3:** Post-operative complications (grade III/IV) and the treatment outcome.

Patient	Complication	Treatment	Outcome
No. 1	Seroperitoneum	Abdominocentesis under local anesthesia	Recovered
No. 2	Hydronephrosis	Ureteral stenting through cystoscope under local anesthesia	Recovered

### 3.3 Follow-up results

The median follow-up time for all retroperitoneal UCD patients was 78.5 months (range, 18–107.5 months). Regular follow-up visits were conducted until the last recorded visit. Encouragingly, all patients remained alive during the follow-up period, and no evidence of disease recurrence was observed. Given the absence of endpoint events, survival analysis was omitted.

## 4 Discussion

Retroperitoneal Castleman disease cases are exceedingly rare worldwide. Existing literature primarily consists of case reports, often involving fewer than two cases (8–11). As a specialized center focused on the surgical management of retroperitoneal sarcomas, we present a comprehensive analysis of unicentric Castleman disease occurring in the retroperitoneum based on a cohort of patients.

### 4.1 Clinical characteristics and diagnostic challenges

Unlike multicentric Castleman disease (MCD), which frequently manifests with symptoms such as polyneuritis, organomegaly, endocrinopathy, and skin changes, most patients with unicentric Castleman disease (UCD) remain asymptomatic except for the localized mass. In our study, only one patient had suspected TAFRO syndrome, which was considered as a special subtype of multicentric Castleman disease (13). This patient presented with splenomegaly and pleural effusion but did not have fever or abnormal hematological markers. The remaining patients showed no significant symptoms, and objective laboratory tests and examinations revealed no abnormalities. This subtle presentation underscores the challenge of early diagnosis (14). In the case of retroperitoneal UCD, patients often lack symptoms until abdominal ultrasound or computed tomography is performed during routine physical examinations (5, 12).

**Figure 1:**
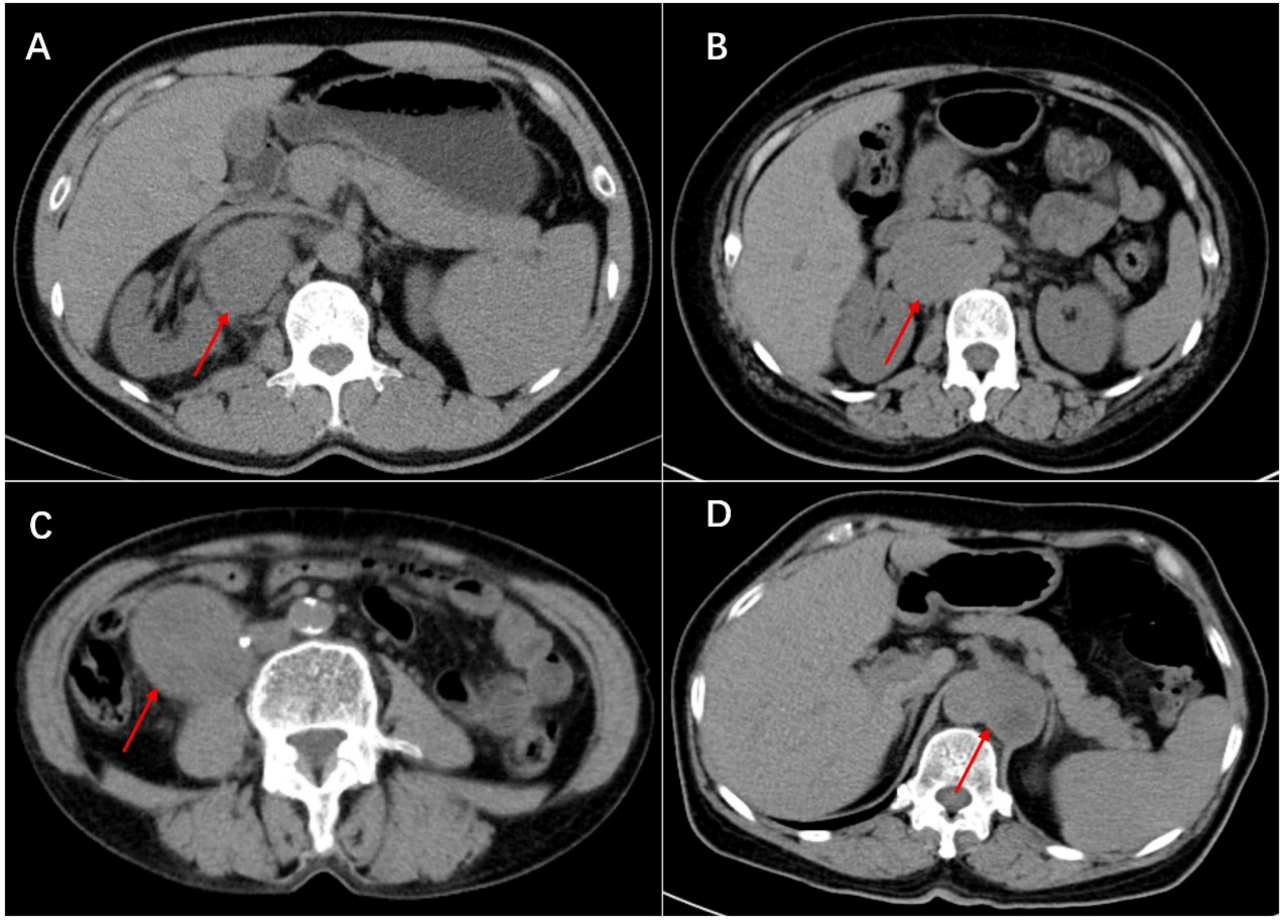
Plain scan of Castleman disease **(A)**, lymphoma **(B)**, leiomyosarcoma **(C)** and paraganglioma **(D)** in enhanced CT scan. Multiple enlarged small lymph nodes can be seen around lesion of Castleman disease and lymphoma. In the image, the leiomyosarcoma can be seen invading the right ureter, resulting in secondary hydronephrosis (not shown in the image), which was managed with a ureteral stent placement before surgery. In particular, splenomegaly can be seen in Castleman disease. They are all cases that have been definitely diagnosed by postoperative pathology.

**Figure 2:**
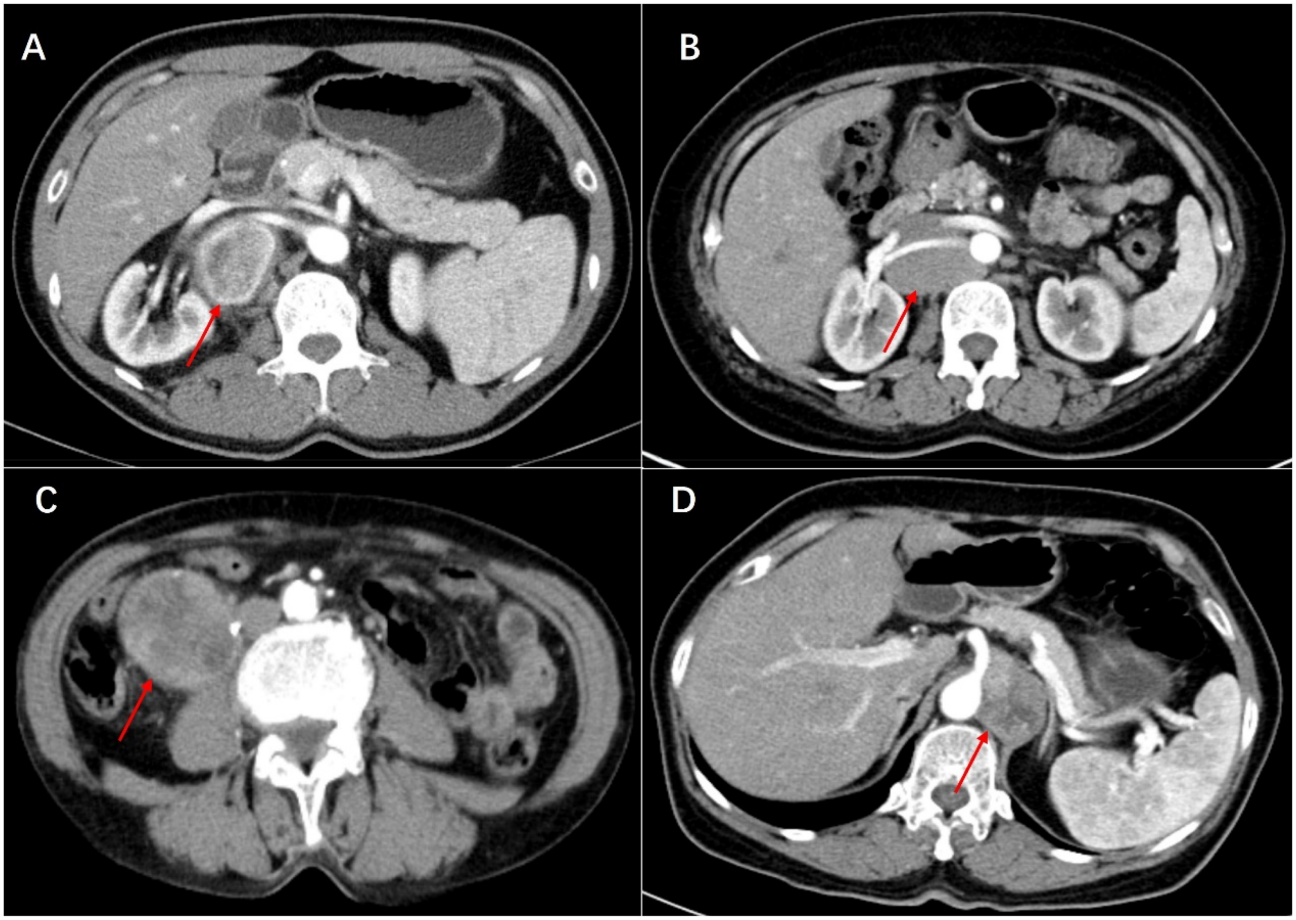
Artery phase of castleman disease **(A)**, lymphoma **(B)**, leiomyosarcoma **(C)** and paraganglioma **(D)** in enhanced CT scan. The tumors show heterogeneous enhancement in the arterial phase. All tumors are closely related to the blood vessels in the retroperitoneum, even encircling the renal artery or celiac trunk.

**Figure 3:**
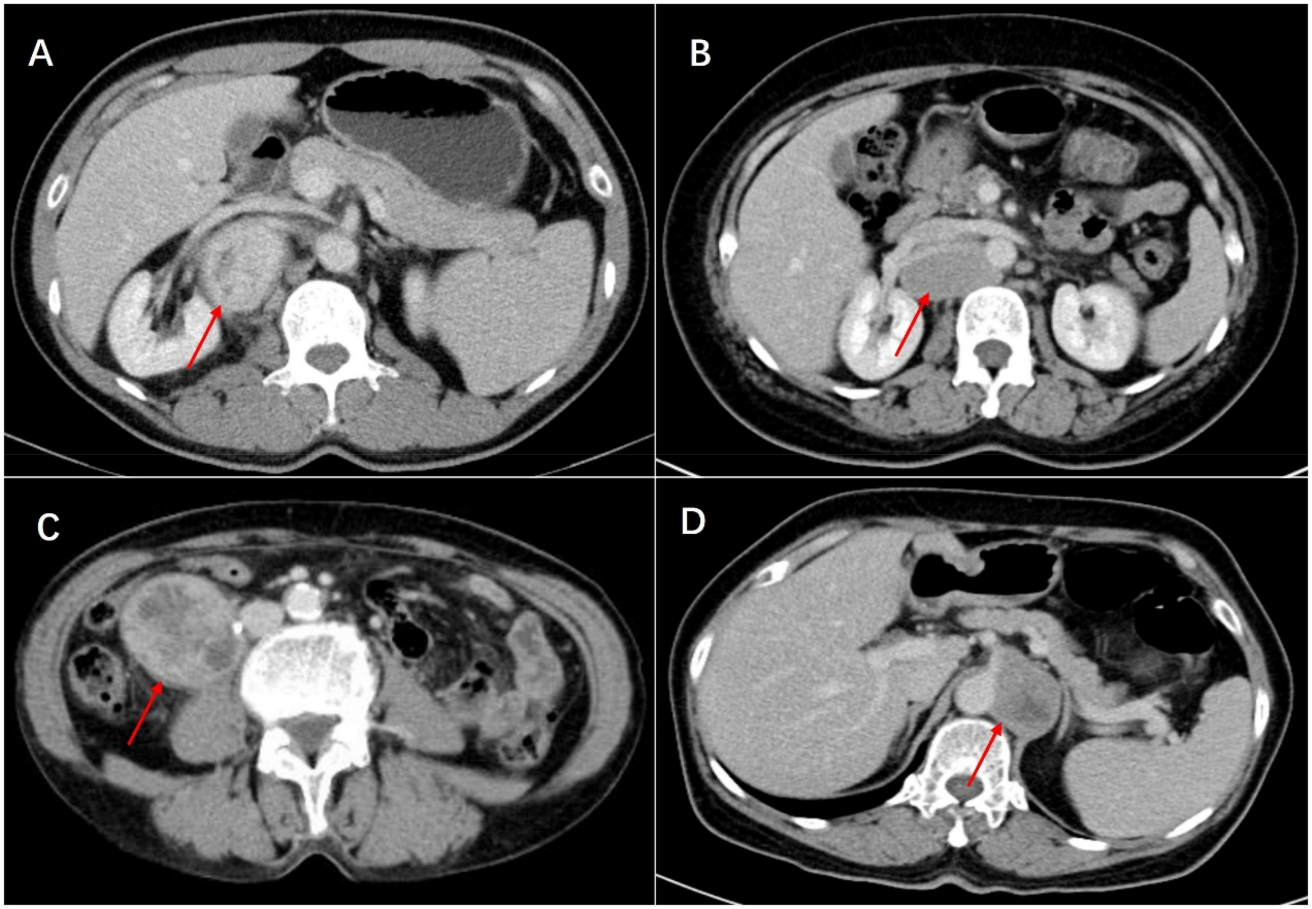
Venous phase of castleman disease **(A)**, lymphoma **(B)**, leiomyosarcoma **(C)** and paraganglioma **(D)** in enhanced CT scan. This lymphoma is highly similar to Castleman disease and surrounds the renal vessels, leading to a high risk of needle biopsy. Ultimately, surgical resection was performed, and postoperative pathology revealed invasive B-cell lymphoma.

Histologically, Castleman disease encompasses three main subtypes: hyaline-vascular (accounting for 90%–91% of cases), plasma cell, and mixed type. The hyaline-vascular subtype is associated with UCD, while the plasma-cell subtype is linked to MCD (6). Definitive diagnosis of retroperitoneal UCD hinges on histological analysis of the mass. However, differential diagnosis remains challenging due to the absence of characteristic symptoms. Preoperative fine-needle aspiration is not recommended due to its low specificity and risk of tumoral seeding (15, 16, 22). Furthermore, fine-needle aspiration has limited utility in CD diagnosis, as it relies on cell architecture rather than cell morphology (15). When encountering patients with isolated retroperitoneal masses exhibiting contrast kinetics along the midline adjacent to the inferior vena cava and abdominal aorta, UCD should be considered, and differential diagnoses should include other highly hypervascular retroperitoneal tumors (e.g., lymphoma, leiomyosarcoma, and paraganglioma, et al.) (see Figures 1–3) (17–19). The imageological distinctions of retroperitoneal UCD and other retroperitoneal tumors are listed in Table 4.

**Table 4:** Imageological distinctions between retroperitoneal UCD and other tumors.

Parameter	rUCD	Lymphoma	Leiomyosarcoma	Paraganglioma
CT	• Uniform enhancement during the arterial phase and sustained enhancement during the venous phase • Multiple small lymph nodes around the lesion	• Multiple enlarged lymph nodes, which may fuse together, forming a mass-like appearance. • Mild to moderate enhancement after contrast administration. • Fused lymph nodes can encase the mesenteric vessels, abdominal aorta, and inferior vena cava, namely distinctive “vascular encasement sign”	• Solitary soft tissue mass • Moderate to high enhancement during the arterial phase, consistent during the venous phase • Typically, no evidence of lymph node metastasis	• Uneven density • Often with hemorrhage, necrosis, calcification and cystic changes • Heterogeneous enhancement in artery phase
PET	• Significant variability in FDG uptake, usually lower than lymphoma • Used to evaluate the number and distribution of lesions in MCD	• Increased uptake of FDG, indicating high metabolic activity • Used to evaluate the number and distribution of lesions	• Increased uptake of FDG • Used to evaluate distant metastasis	• Increased uptake of FDG • Used to evaluate distant metastasis

rUCD, retroperitoneal unicentric Castleman disease; FDG, fluorodeoxyglucose; MCD, multicentric Castleman disease; CT, computed tomography; PET, positron emission tomography.

### 4.2 Surgical approach and prognosis

Complete surgical resection remains the gold standard for treating UCD, including retroperitoneal UCD. The prognosis for retroperitoneal UCD is generally favorable. Most patients achieve long-term survival following R0 resection. Systematic reviews indicate that complete resection alone, without additional treatment, yields excellent outcomes, with 5-year disease-free survival (DFS) rates exceeding 80% and overall survival (OS) rates surpassing 90% (5, 6).

In our study, all 15 patients with retroperitoneal UCD underwent complete resection of the primary lesion as the initial treatment, without additional therapies. Remarkably, all patients remained alive during the follow-up period, and no evidence of disease recurrence was observed. Although our patient cohort was small, our surgical strategy—favoring extended resection margins to ensure radical cure—may have contributed to these positive outcomes. This finding underscores the importance of radical resection with negative margins in patients suspected of having UCD but lacking definitive diagnosis. Longer follow-up and larger patient cohorts are needed to validate the impact of extended resection on retroperitoneal UCD. Currently, no standardized follow-up protocol exists for resected UCD. Based on existing literature, we recommend CT scans every 6 months during the first 3 years postoperatively, followed by annual scans thereafter.

### 4.3 Surgical challenges and strategies

Our experience highlights the significant challenge posed by intraoperative bleeding during resection of retroperitoneal UCD. The adjacent and surrounding blood vessels, primarily branches of the abdominal aorta and inferior vena cava, contribute to this complexity. To mitigate operative risks and ensure safety, comprehensive radiographic examinations play a crucial role. These examinations should include:

• Color Ultrasonography: Provides real-time visualization of blood flow patterns and helps assess vascular relationships.
• Contrast-Enhanced Computed Tomography (CT): Offers detailed anatomical information, aiding in precise evaluation of lesion-to-vessel proximity.
• Angiography (if necessary): Allows direct visualization of vascular structures and assists in surgical planning.

By meticulously assessing the relationship between lesions and adjacent vessels, surgeons can navigate the retroperitoneal space safely. Notably, laparoscopic surgery emerged as a viable option for selected patients. In our cohort, two patients underwent laparoscopic procedures without perioperative complications, and long-term follow-up revealed no recurrences.

### 4.4 Study limitations

First, as a retrospective study, inherent biases in patient selection and data collection may exist. Second, Patients were often screened by other hospitals and departments before seeking our specialized team's expertise, potentially introducing additional selection bias. Besides, the rarity of retroperitoneal UCD limited the number of patients available for final analysis. Due to the small sample size, we could not directly compare different treatment strategies (e.g., incomplete resection vs. radiotherapy). As patients were initially managed as malignant sarcomas, certain Castleman disease-related details (e.g., human herpes virus 8 status, serum immunoglobulin G, interleukin-6 levels) were lacking (20, 21).

## 5 Conclusion

Our findings underscore that complete resection remains the gold standard for treating retroperitoneal UCD. Achieving excellent survival outcomes with minimal surgery-related morbidity validates this approach. Furthermore, experienced surgeons can safely explore laparoscopic surgery in carefully selected patients. Future studies should validate our results and deepen our understanding of this rare disease.

## Data Availability

The raw data supporting the conclusions of this article will be made available by the authors, without undue reservation.
